# Supplementary material for: Individual and community-level determinants of underweight among lactating mothers in Ethiopia: A multilevel analysis
Source: PLoS One. 2022 May 5;17(5):e0267821. doi: 10.1371/journal.pone.0267821 (PMC9070908; doi:10.1371/journal.pone.0267821)
Supplement: Attachment — Submitted filename: Response to Reviewers.docx [file pone.0267821.s002.docx]

| **Commented by** | **Comments raised** | **Authors’ response** |
| --- | --- | --- |
| Editor | Please ensure that your manuscript meets PLOS ONE's style requirements, including those for file naming. | Thank you very much for the feedback. We have revised the manuscript outline as per the PLOS one’s style. |
| Editor | We noticed you have some minor occurrence of overlapping text with the following previous publication(s) which needs to be addressed: https://bmjopen.bmj.com/content/10/3/e034963  The text that needs to be addressed involves parts of the Discussion and Conclusions.  In your revision ensure you cite all your sources (including your own works), and quote or rephrase any duplicated text outside the methods section. Further consideration is dependent on these concerns being addressed**.** | - Thank you very much for the feedback. We have made corrections to the manuscript files according to your comment. - we have cited all sources used in this manuscript and ,also paraphrased overlap sentences |
| Editor | Please review your reference list to ensure that it is complete and correct. If you have cited papers that have been retracted, please include the rationale for doing so in the manuscript text, or remove these references and replace them with relevant current references.  Any changes to the reference list should be mentioned in the rebuttal letter that accompanies your revised manuscript. If you need to cite a retracted article, indicate the article’s retracted status in the References list and also include a citation and full reference for the retraction notice | Thank you very much for the feedback. We have checked all of the references for correctness and there are no retracted references. |
| Editor | Please include a paragraph on following two key terminologies in methods section. 1) Community poverty: This needs a clear description. | Thank you for valuable suggestion and comments. We tried to clearly describe “community-poverty” in the revised manuscript. Thanks once again. |
| Editor | 2) Multilevel analysis: The basis of selecting multilevel analysis. How was the model built? Is it based on a priori hypothesis? | Thank you very much for your valuable concerns; we have selected multilevel analysis Since EDHS data are hierarchical, i.e., mothers are nested within households, and households are nested within clusters, the use of standard models could underestimate standard errors of the effect sizes, consequently affecting the decision on the null hypothesis. With such data, mothers within a cluster may be more similar to each other than mothers in the rest of the cluster. This violates the assumption of the standard model; independence of observation and equal variance across the cluster which implies a need to consider the variability between clusters. All these issues are motivated us to use the multilevel modeling. We stated these concepts under method part of data management and analysis. Thanks once again for your concerns! |
| Editor | 3) Please include a STROBE check list. | Thank you for your comments; we have now incorporated the STROBE cross-sectional checklist in the revised manuscript. We have also uploaded the filled checklist as supplementary file. |
| Reviewer 1 | Have the authors made all data underlying the findings in their manuscript fully available? #NO | Thank you for your comments; we used secondary data of EDHS. The data that support the findings of this study are available from the Measure DHS project upon reasonable request. Permission to use the 2016 EDHS database for further analysis can be sought from http://www.dhsprogram.com |
| Reviewer 1 | Good quality article using secondary data. Can be further used as hypothesis for testing by planning an analytical study to prove the causal association of the variables identified. The study needs to be uptaken for intervention and policy planning and recommendations | Thank you very much. we have further improved quality of the manuscript as per yours’ and other reviewers comments. |
| Reviewer 2 | Paper is technically fine. analysis is fine and in line with study objectives. Study also opens the door for future research in nutrition sector in Ethiopia. However, correlation and synchronization with programmatic updates will make it more useful. | Thank you very much. we have further improved quality of the manuscript as per yours’ and other reviewers comments. |
